# The Association between Primary Endometrioid Carcinoma of the Ovary and Synchronous Malignancy of the Endometrium

**DOI:** 10.1155/2010/465162

**Published:** 2009-12-15

**Authors:** Catharina C. van Niekerk, Johan Bulten, G. Peter Vooijs, André L. M. Verbeek

**Affiliations:** ^1^Department of Epidemiology, Biostatistics, and Health Technology Assessment, Radboud University Nijmegen Medical Centre, P.O. Box 9101, 6500 HB Nijmegen, The Netherlands; ^2^Department of Pathology, Radboud University Nijmegen Medical Centre, 6500 HB Nijmegen, The Netherlands; ^3^Institute of Technical Medicine, University of Twente, 7500 AE Enschede, The Netherlands

## Abstract

*Objective*. Ovarian and endometrial cancers coincide rather frequently in the same patient. Few data are available on the involvement of the specific morphological subtypes. To identify histological pathways in the synchronous occurrence, a population-based study was performed in The Netherlands. *Methods*. Using the national pathology database (PALGA) information of ovarian cancers and of earlier or later cancer in the endometrium was obtained. 5366 Patients were identified with primary malignant epithelial or borderline malignancy. *Results*. In 157 cases (2.9%) a new primary malignancy in the endometrium was diagnosed (146 within 1 year). The ratio of observed versus expected number of synchronous malignancy in the endometrium was estimated at 3.6 (95% CI: 2.7–4.7). Among 460 ovarian endometrioid carcinoma patients 53 cases showed a second primary endometrial cancer; 40 out of these 53 cases (75.5%) showed at both organ sites an endometrioid adenocarcinoma. *Conclusion*. These findings suggest an important role for the endometrioid subtype and prompt to mechanism-based studies incorporating molecular techniques.

## 1. Introduction

Approximately 10% of all patients with ovarian cancer appear to have endometrial cancer synchronously, and 5% the other way around [1]. However, it is often unclear whether this confers to primary tumors or to metastasis from the ovary to the endometrial tumor or vice versa [2, 3]. As described by Herrinton et al., both of these tumors are probably mechanistically linked to reproductive hormones. But it is also possible that the joint presence of these two tumors in different organ sites indicates to etiologically distinct and until now unknown conditions [4].

According to the sparse literature, and which mainly consists of case series, the simultaneous presence of primary cancers in the ovary and the endometrium is not well documented. A strong association has first been quantified by Sheu et al. [5]. More recently, Van Niekerk et al. [6] calculated for ovary cancer the observed versus expected numbers of cancer in the endometrium to be a ratio of 62.3. This strong relationship prompted us to further evaluate the risk by histological subtype of the epithelial ovarian tumors.

Most ovarian tumors are adenocarcinomas of different histological subtypes, derived from the surface epithelium of the ovary [7]. They manifest in various morphological forms as (cyst)adenocarcinomas with serous, mucinous, clearcell, or endometrioid differentiations. Further to that it is known that primary endometrial neoplasms include the same subtypes [8].

## 2. Material and Methods

### 2.1. Design and Patients

We examined the association of the various histopathological subtypes of ovarian epithelial cancer in relation to second primary endometrial cancers using two random samples of the nationwide pathology database “PALGA” in The Netherlands. Every record in the PALGA database contains a summary of the full pathology report and diagnostic codes similar to the Systematized Nomenclature of Medicine (SNOMED) classification of the College of American Pathologists. From the first sample of the years 1987–1993 we investigated 4577 patients and from a second sample of the years 1996–2003, a number of 789 cases [6]. Of these 5366 patients with a new malignant or borderline malignant epithelial ovarian cancer diagnosis we also obtained all the other histopathologically confirmed diagnoses of primary invasive malignancies in the endometrium if present, and earlier than, concurrently or after the ovarian tumor was diagnosed.

The scientific committee of PALGA approved the study protocol beforehand.

### 2.2. Measurements

Erroneous coding of the pathologist can hamper the interpretation of diagnostic codes. Therefore, we also studied a second more recent and smaller PALGA dataset. The diagnostic codes in the PALGA database were reviewed, and the corresponding pathology conclusions, that is, PALGA codes and PALGA conclusions, as well. Two experienced pathologists (GPV, JB) reviewed all reports.

The criteria of Young and Scully [8] were used for interpretation of synchronous primary tumors of both organs or of metastasis from one organ to the other. The diagnosis of independent primary tumors could be made in most cases. Histological dissimilarity of the tumors at both organ sites makes two independent synchronous tumors highly probable. In addition, if the codes and conclusions report no or only superficial myometrial invasion of the endometrial tumor and/or both tumors were confined to the ovary and uterus, the diagnosis of two independent primary tumors could be reliably made.

If it was doubtful whether or not we were dealing with a metastasis or recurrence of the primary malignant tumor of the ovary or a new secondary type of tumor, these uncertain diagnosis and difficult cases were excluded.

### 2.3. Data Analysis

Descriptive analysis was applied to the ovarian epithelial cancers for histopathological subtypes. For the major histological subtypes the number of patients observed with a second primary endometrial cancer was contrasted to the expected number. Expected numbers were calculated from the 5-year age specific rates of 2nd primaries in the total ovary cancer group. The observed versus expected ratio and its 95% confidence interval (CI) were calculated according to the method of Byar [9].

## 3. Results

157 (2.9%) cases of the reviewed 5366 patients with ovarian epithelial cancer appeared to have a second primary malignant tumor in the endometrium (146 within 1 year). In both samples this percentage was identical (2.9%). The mean age at diagnosis of all patients with ovarian cancer was 59.6 years; the 157 cases aged 58.6 years on average. The histological subtypes and age results are shown in Table 1. In general, three quarters of all patients aged 50 years and over, except for patients with a mucinous tumor, of whom almost half of them are under the age of 50.

If a second primary cancer is present in the endometrium, the endometrioid carcinoma subtype in the ovary is found to be more frequent in the younger age-group (<50), while mucinous and serous cancer are less frequent.

**Figure 1:**
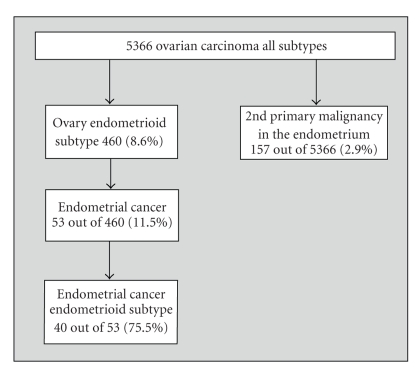
Number of second primary tumors in the endometrium in 460 patients with endometrioid ovary cancer.

As can be derived from Figure 1, the relative frequency of endometrioid carcinoma is 8.6%, and also highly prevalent if a second primary is present in the endometrium (*n* = 53, 11.5%). We observed that 40 out of these 53 cases (75.5 %) had an endometrioid adenocarcinoma in the ovary as well as in the endometrium.

In Table 2, the observed versus expected numbers of cancer of specific histology are presented among the 157 cases, having both ovary cancer and a second primary cancer of the endometrium. For the 53 (33.8%) cases with endometrioid cancer in the ovary the observed versus expected ratio was calculated to be 3.6 (95% CI: 2.7–4.7), implicating a more than threefold risk of second primary in the endometrium. The other histological subtypes did not reveal such an excess risk, but a lower finding (mucinous and serous carcinoma).

## 4. Discussion

The present study with data from the PALGA nation-wide pathology archives in The Netherlands reports a strong association between the occurrence of epithelial malignancy in the ovary of the endometrioid histological subtype and a second primary malignancy in the endometrium. The study period was comparable with the study of Vernooij et al., also from The Netherlands [10]. The latter nationwide study focused on survival of patients with ovarian cancer and hospital type. The percentages for the different histological subtypes of ovarian carcinomas (Table 1) are very much concordant across both study groups [10]. Only a slight difference in the “other” and “adenocarcinoma” categories was noticed. Precise percentages about the histological subtypes of ovarian carcinomas are hardly found in the international literature and gynaecopathological leading handbooks. The reason is that most referred studies are often small, have incomplete data, and are difficult to compare. Moreover, if histological subtypes are given, the percentages in literature and overviews mostly also include benign ovarian neoplasm's [11].

We found in both databases, 1987–1993 and 1996–2003, a similar 2.9% incidence of synchronous primary ovarian and endometrial cancer. This is in accordance with the study of Chiang et al. [1] and Williams et al. [12]. The objective of their study was to clarify the potential factors that influence the survival of patients with simultaneous primary malignancies in the endometrium and ovary. The group of Chiang [1] retrospectively reviewed the medical records and pathologic reports from the National Taiwan University Hospital Cancer Registry from the period 1997–2005. They detected 27 cases out of 1004 (2.7%) ovarian carcinoma patients with a malignancy of the endometrium as well. Williams et al., [12] identified 1.355 synchronous ovarian and endometrial cancer cases in a total of 56.986 primary ovarian cases (2.4%) diagnosed in the period 1973–2005. They used the SEER definition of synchronous cancers, that is, cancer of the endometrium (C54.1) and ovary (C56.9). Women were excluded from analysis if they were diagnosed with other primary cancers (e.g., from breast, colon, or cervix).

Recently, the simultaneous presence of primary cancers in the ovary and the endometrium has been quantified by Soliman et al. [2], and by Hemminki et al. [7, 13]. They also specified the relation to the histological subtype of the ovarian tumor. The median age in our study and in Soliman's study was almost similar at 53 and 50 year, respectively, for the endometrioid carcinomas. In our study 53 (33.8%) out of 157 patients having a second malignancy in the endometrium had an endometrioid malignancy in the ovary, while 40 (75.5%) out of those 53 cases also showed an endometrioid subtype in the endometrium. The study of Soliman reported 57 (68%) individuals with endometrioid malignancy in the ovary and endometrium out of 84 cases having indepent primary cancers in both of these organs. It remains unclear, however, from how many patients with ovarian malignancy these 84 women originated.

Hemminki et al. [7] reported an age-standardized incidence ratio of SIR = 86.7, (95% CI: 46.0–148.6) for ovarian endometrioid cancers and simultaneous primary endometrial carcinoma (13 cases). The difference in outcome with our investigation may be due to the character of their database, the Swedish Family Cancer-Database, which differs from our national pathology database of all cancers and not discerning family background.

In a different study Hemminki and Granström [13] describe a strong link of familial ovarian and endometrial cancers, which appears to be specific for the endometrioid morphology. They calculated an SIR = 3.40, (95% CI: 1.80–5.83), implicating a 3.4-fold risk of endometrioid ovarian cancer among daughters of mothers presenting endometrial cancer. Unfortunately, our database did not contain family background information.

Endometrioid adenocarcinoma is the most common type of endometrial adenocarcinonomas occurring in more than three-quarter of all cases [14]. Hyperestrogenic status plays an essential part in the origin of this subtype of endometrial carcinoma, as most of these patients have complaints of irregular menses, infertility, obesity, and polycystic ovary disease. As described by Chiang et al., [1] the pathogenesis of synchronous endometrial and ovarian cancer is unclear. The theory of a secondary Müllerian system says that the epithelium of cervix, uterus, fallopian tubes, ovaries, and peritoneal surface have shared molecular receptors responding to carcinogenic stimulus leading to the development of multiple primary malignancies synchronously. They further describe that the hypothesis provides an explanation for synchronous malignancies of similar histology. This may not be the case in synchronous cancers of dissimilar histology, and there a different mechanism underlying this interesting phenomenon should be operating. Further studies are needed to disclose the pathogenesis of synchronous ovarian and endometrial cancer.

Halperin et al. [15] compared 16 cases of simultaneous independent primaries of endometrium and ovary, presenting the same histological subtype, and 12 cases of primary endometrial cancer demonstrating ovarian metastases. The only clinical parameter differentiating significantly between the groups was the prevalence of familial cancer, being more frequent in the group of metastatic tumors. They further notified that the application of immunohistochemical analysis of estrogen and progesterone receptors is of value in the differentiation between cases of simultaneous independent carcinomas of endometrium and ovary versus cases of endometrial carcinoma with ovarian metastasis. We believe that immunohistochemical protein analysis will probably not discriminate for the same primary epithelial morphological subtypes arising in different organs. It is expected that concomitant tumors with exactly the same morphology arising in ovary and endometrium, especially the endometrioid carcinoma, will show the same immunohistochemical expression patterns.

Molecular markers emerging as mutation from PTEN and LOH analysis as described by Ricci et al. [16] may be more suitable to establish a correct final diagnosis in distinguishing between metastasis from primary synchronous carcinomas of the endometrioid subtype of the ovary and endometrium. The potential of these molecular markers has to be evaluated in larger series, because so far this has been done in only few patients [16, 17].

In the study of Soliman et al., 7 (7%) out of 102 women with synchronous endometrial and ovarian cancer had either clinical or molecular criteria suggestive for Lynch syndrome [18]. They believe that genetic evaluation of women with synchronous ovarian and endometrial cancer who had a prior history of at least one first-degree relative with an HNPCC-associated cancer may appropriately be identified as women with Lynch syndrome.

In summary, our results indicate that in 2.9% of patients diagnosed with epithelial ovarian malignancy a second new primary malignant tumor is occurring in the endometrium, especially in women diagnosed with an endometrioid histological subtype (33.8%). This histological tumor subtype is most prevalent in the age category of 50–54 years (30.2%) and shows a ratio of observed versus expected number of cases with a for endometrium malignancy being 3.6 (95% CI: 2.7–4.7).

Endometrioid adenocarcinoma at both organs sites is by far out the most prevalent subtype (75.5%). The other histological subtypes do not reveal such excess risks. These findings should stimulate further molecular studies into the possibly carcinogenic pathways.

## Figures and Tables

**Table:** (a) All epithelial ovarian cancers according to histological type and age at diagnosis.

Histological type of ovarian cancers	Patients *n*	%	Range	Median	Mean	% <50 years	% ≥50 years
Adenocarcinoma	1456	27.1	18–97	64	62.5	16.3	83.7
Clearcell carcinoma	236	4.4	28–88	58	59.2	23.7	76.3
Endometrioid carcinoma	460	8.6	20–86	59	58.8	25.4	74.6
Mucinous carcinoma*	733	13.7	14–92	52	52.1	44.9	55.1
Serous carcinoma*	1801	33.6	16–100	62	59.9	22.9	77.1
Others^∗a^	680	12.7	16–100	62	61.5	20.9	81.2

Total	5366	100	14–100	61	59.6	24.2	75.8

**Table:** (b) Subset of epithelial ovarian cancers with malignancy in the endometrium.

Histological type of ovarian cancers	Patients *n*	%	Range	Median	Mean	% <50 years	% ≥50 years
Adenocarcinoma	49	31.2	34–85	59	59.8	22.5	87.5
Clearcell carcinoma	3	1.9	53–78	71	67.3	0.0	100.0
Endometrioid carcinoma	53	33.8	36–80	53	54.9	32.1	67.9
Mucinous carcinoma	7	4.5	49–73	57	59.7	14.3	85.7
Serous carcinoma*	27	17.2	31–78	68	64.1	11.1	88.9
Others^a^	18	11.5	46–78	55	57.9	22.2	79.8

Total	157	100	31–85	57	58.6	22.9	77.1

*borderline malignancies included.

^a^including mixed carcinomas, anaplastic carcinoma, malignant Brenner tumor, carcinosarcoma, adenosquamous carcinoma, squameus cell carcinoma etc.

**Table 2:** Association among 157 cases between subtype of epithelial ovarian cancer and second endometrial cancer.

Histology of ovarian tumors	Observed	Expected	O/E**	95%-CI
Adenocarcinoma	49	42.82	1.14	(0.85–1.51)
Clearcell carcinoma	3	7.57	0.40	(0.08–1.16)
Endometrioid carcinoma	53	14.93	3.55	(2.66–4.64)
Mucinous carcinoma*	7	19.30	0.36	(0.15–0.75)
Serous carcinoma*	27	52.62	0.51	(0.34–0.75)
Others	18	19.75	0.91	(0.54–1.44)

*including borderline malignancies.

**Observed versus Expected number of cases of synchronous endometrial cancer.
